# Is reduced self-esteem a necessary condition for eating disorder symptoms in adolescence? Preliminary evidence from a necessary condition analysis study

**DOI:** 10.1007/s40519-025-01806-4

**Published:** 2025-12-18

**Authors:** Igor Marchetti, Ilaria Colpizzi, Elide Francesca De Caro, Lavinia Miriam Pedretti, Sara Iannattone, Lisa Di Blas, Gioia Bottesi

**Affiliations:** 1 Dipartimento di Scienze della Salute (DSS), Università di Firenze, Florence, Italy; 2 Department of Life Sciences, University of Trieste, Via Edoardo Weiss 21, 34128 Trieste, Italy; 3 Department of Philosophy, Social Sciences and Education, University of Perugia, Perugia, Italy; 4 Department of General Psychology, University of Padua, Padua, Italy

**Keywords:** Necessary conditions, Eating disorders, Adolescence, Self-esteem, Necessary conditions analysis

## Abstract

**Purpose:**

Eating disorder (ED) symptoms are common psychopathological manifestations, with significant impacts on quality of life, particularly among female adolescents. Despite the high mortality rates of full-blown EDs (5–20%), the mechanisms underlying vulnerability remain poorly understood. Traditional approaches primarily examine probabilistic sufficient factors (i.e., regression coefficients); however, these models fail to accurately predict ED symptoms onset in non-clinical populations. This study shifts focus to necessary conditions—factors whose absence precludes the development of the outcome—using necessary condition analysis (NCA), a novel methodological approach.

**Methods:**

We examined whether lower self-esteem is a necessary condition for restriction-oriented cognitions (*Drive for Thinness*), dissatisfaction about one's body (*Body Dissatisfaction*), and dysregulated eating behaviors (*Bulimia*) in female adolescents (*N* = 84; mean age = 15.74 ± 1.30 years) after 12 months.

**Results:**

Results preliminarily indicated that lower self-esteem emerged as a necessary condition for restriction-oriented cognitions (*d* = 0.25, *p* < 0.003) and for dissatisfaction with the body (*d* = 0.22, *p* < 0.003). However, it was not a necessary condition for dysregulated eating behavior.

**Conclusions:**

These findings highlight the potential of NCA to refining theoretical models and clinical interventions by distinguishing necessary conditions from sufficient factors. The study underscores the importance of integrating necessity logic into ED research, offering insights for targeted prevention and personalized care.

*Level of evidence*: Level III: Evidence obtained from well-designed cohort or case–control analytic studies.

## Introduction

Eating disorders (EDs) are severe psychiatric conditions characterized by a distorted body image and maladaptive eating behaviors, significantly impacting quality of life and overall well-being [1]. EDs particularly affect women, with lifetime prevalence rates ranging from 5.5% to 17.9% among adolescent and young adult females [2]. The incidence of EDs increases in female adolescents with a peak in late adolescence, highlighting the importance of this life stage [3]. Similarly, subclinical ED symptoms can lead to levels of impairment comparable to those observed in full-blown disorders, yet they affect a significantly larger proportion of women than clinically diagnosed EDs [4]. Hence, expanding our understanding of the underlying mechanisms contributing to vulnerability and enhancing assessment processes is paramount.

Although previous research has identified numerous risk factors across genetic, environmental, social, developmental, and psychological domains [5], screening interventions in non-clinical populations remain insufficient for accurately predicting the onset of ED symptoms [6]. This challenge may partially stem from the reliance on traditional analytical approaches—such as multiple regression models and structural equation modeling—commonly used to predict the development and trajectory of ED symptoms. These methods are based on the premise that a high regression coefficient indicates that an increase in a predictor variable is linked to a higher likelihood of the outcome occurring. Consequently, in causal terms, these models aim to identify probabilistic sufficient factors ("if X, then *probably* Y") [7].

Probabilistic sufficient factors exhibit two key characteristics. First, these factors are not a prerequisite and can be replaced [8]. In other words, if one predictor is absent or of negligible magnitude, other factors can compensate, leading to similar outcomes. For example, lower levels of preoccupation with food might be offset by higher levels of perfectionism, still leading to the development of ED symptoms. Second, probabilistic sufficiency offers insights into what is likely to occur on average across a group of cases (i.e., the average effect) [7], rather than predicting outcomes at the individual level. This feature limits the ability of probabilistic sufficient factors to accurately predict an individual's trajectory in developing future psychopathology [9].

While these approaches have offered valuable contributions, their methodological features may limit the effectiveness of detection of ED symptoms in certain contexts, posing challenges across various settings (e.g., primary care and healthcare) and populations (e.g., university students, individuals with comorbidities, and children) [6]. To complement these approaches, recent work has proposed broadening the conventional sufficiency-focused framework by also incorporating perspectives based on necessity logic [7–9]. A necessary condition is a factor whose presence enables the possibility of an outcome without ensuring it (“if X, then *maybe* Y”), while its absence guarantees the absence of the outcome (“if not X then *always* not Y”) [7]. Notably, the absence of such a necessary cause consistently indicates the non-occurrence of the outcome at individual level, aligning with *deterministic necessity logic* rather than probabilistic. Furthermore, failing to meet a necessary condition renders the outcome impossible, and this condition cannot be substituted by the fulfillment of other necessary conditions. Importantly, this characteristic makes the identification of a single necessary condition highly informative on its own, without the need for complex models to understand vulnerability to psychopathology. For instance, breathing is a necessary condition for human survival; its absence always leads to death, regardless of whether other necessary conditions for life, such as drinking water, are met.

In 2016, Dul introduced necessary condition analysis (NCA), a novel method for identifying, quantifying, and drawing statistical inferences about necessary conditions. This approach has been successfully applied in various fields, including economics, public health, and business management [8, 10]. While NCA’s application in the fields of clinical psychology and psychiatry has been limited until recently [11], Marchetti et al. [12] have highlighted its potential in adolescent mental health research. In a study of 382 adolescents, they evaluated cognitive and personality traits, symptom severity, stressful life events, and depression history at baseline. Over a 2-year follow-up, they tracked the onset of major depressive episodes (MDEs), revealing that baseline depressive symptoms, rumination, self-criticism, and stressful experiences were necessary conditions for MDEs, while other factors, such as dependency traits, dysfunctional attitudes, and hopelessness, were not necessary. Following this study, Colpizzi and colleagues [13] found that several cognitive vulnerability factors were necessary conditions for the development of future depressive symptoms in adolescent girls, but not in boys. Along these lines, another study showed that intolerance of uncertainty was a necessary condition for the development of subclinical levels of various types of anxiety symptoms in adolescents over a 6-month period [14].

In the context of assessing ED symptoms risk in youth, the NCA method holds significant promise for identifying individuals who are virtually immune to developing ED symptoms, distinguishing them from those who may be at risk. This approach has the potential to isolate a subset of necessary factors from the broader spectrum of vulnerability factors [12], improving our understanding of ED symptoms and informing early detection, prevention strategies, and clinical interventions. Furthermore, within a deterministic framework, NCA studies can offer valuable insights that can be applied from group-level findings to individual cases, supporting personalized care [9].

In this study, we focused on one of the most widely recognized risk factors for ED symptoms, namely, low self-esteem [15, 16]. Low self-esteem is considered one of the core elements of eating pathology in several developmental and maintenance theories of ED symptoms [17], particularly in Fairburn and colleagues' model [18], in which this factor reflects dissatisfaction with oneself and, consequently, with one’s body. For individuals with or vulnerable to EDs, self-esteem is often closely tied to their perceived inability to control food, weight, and body shape, which can drive them to engage in extreme eating behaviors in an attempt to improve their self-image [18].

Accordingly, we investigated whether lower self-esteem constitutes a necessary condition for the emergence of core ED symptoms. These encompass both cognitive–affective symptoms—cognitions related to restrictive tendencies (*Drive for Thinness*) and body dissatisfaction (*Body Dissatisfaction*)—and behavioral symptoms, namely, dysregulated eating behaviors (*Bulimia*) [19], in a non-clinical sample of female adolescents aged 13 to 19, assessed over a 12-month period. Adolescence represents a critical stage for both self-esteem and ED symptom development, as peer comparisons strongly shape self-perception [20], and ED onset typically peaks between ages 15 and 16 [21]. Because NCA is a novel method for examining necessity relations between predictors and outcomes, the study was primarily exploratory, and no hypothesis was pre-registered. Nonetheless, we expected that lower self-esteem would emerge as a necessary condition for higher levels of ED-core symptoms. Our overarching aim was thus to provide preliminary evidence for the utility of NCA as an innovative framework to clarify the role of vulnerability factors in ED research.

## Methods

### Study design and participants

The present longitudinal study consisted of 84 female adolescents (ages 13–19 years; *M* = 15.74, *SD* = 1.30) attending I.T.E.T. “G. Salvemini” in Molfetta (Bari, Italy), a public, secondary technical school. The school serves approximately 510 students across 31 classes (~ 16 students per class), indicating a small-sized institution. The student body is predominantly lower-middle socioeconomic status; many households are single income (often with a stay-at-home mother), with a notable proportion of unemployed parents. About 10% of students hold non-Italian citizenship. Post-secondary continuation is low and early school leaving is a recurrent risk. Data were collected between spring 2017 and spring 2019. Follow-up assessment was conducted exactly 12-month post-baseline.

At baseline, the participants had a mean Body Mass Index (BMI) of 21.21 (SD = 2.94). Adolescents were asked to complete a series of self-report questionnaires designed to assess various psychological, behavioral, and health-related factors at baseline and again after 12 months. Over the course of the study, between 2 and 6 participants either discontinued their participation or provided incomplete data during follow-up assessments. A priori NCA power analysis indicated that to detect a moderate-to-large effect size (*d* = 0.20) with a α level of 0.05 and a statistical power of 0.80, a minimum sample size of 76 participants would be required. This sample had previously been used previously [22] and the study was not pre-registered.

### Measures

#### Self-esteem

The *Rosenberg Self-Esteem Scale* (RSES [23]) is a widely used instrument designed to assess individual self-esteem. It consists of 10 items, such as " I feel that I am a person of worth, at least on an equal plane with others" rated on a four-point Likert scale. Response options range from 1 (Strongly Disagree) to 4 (Strongly Agree), with higher scores reflecting greater levels of self-esteem. In the current sample, Cronbach’s alpha coefficient was 0.82.

#### ED vulnerability

The Eating Disorder Inventory-2 (EDI-2 [24]) is a 91-item self-report questionnaire widely used in both clinical and research contexts to assess psychological traits and behavioral patterns commonly associated with EDs. It comprises 12 subscales, including *Drive for Thinness, Body Dissatisfaction*, and *Bulimia* which target attitudes related to eating, weight, and body image, as well as subscales, such as *Ineffectiveness, Interpersonal Distrust,* and *Perfectionism*, which address broader psychological characteristics. In the present study, only the *Drive for Thinn*ess (“I am preoccupied with the desire to be thinner”), *Body Dissatisfaction* (“I think that my stomach is too big”), and *Bulimia* (“I have gone on eating binges where I have felt that I could not stop”) subscales were analyzed. In the current sample, Cronbach’s alpha coefficients were 0.87 for *Drive for Thinness*, 0.83 for *Body Dissatisfactio*n, and 0.62 for *Bulimia*, aligning with reliability indices reported for the Italian normative sample [25].

As the EDI-2 does not provide standardized cutoff scores to distinguish clinical from non-clinical populations, percentile-based thresholds were applied. Subclinical levels of symptoms were defined as scores equal to or above the 95th percentile of the Italian normative data, specifically ≥ 19 for *Drive for Thinness,* ≥ 20 for *Body Dissatisfaction*, and ≥ 8 for *Bulimia* [25]*.* These thresholds are broadly consistent with Garner’s [25] recommendation that scores above the 94th percentile may indicate subclinical symptoms.

### Data analysis approach

The NCA visualizes data through scatterplots, mapping predictor values against outcome values to define an area of empirically possible observations, called the *scope* [8]. It also identifies a region with virtually no data points, referred to as the *ceiling or empty zone*, found in the upper-left corner of the plot, where a high value of X is necessary for a high value of Y to occur. Alternatively, the ceiling zone located in the upper-right corner, where a low value of X is necessary for a high value of Y to occur. The empty zone indicates that the absence of the predictor is necessary for the absence of the outcome, meaning that if the predictor does not reach a certain level, the outcome cannot occur. However, even if the predictor meets the required level, the outcome is possible but not guaranteed. Conversely, the absence of the required level of the predictor ensures the absence of the level of the outcome [8].

Among the techniques available for defining the ceiling zone, the main method is the ceiling envelopment–free disposal hull (CE–FDH [8]). The CE–FDH method generates a non-decreasing, piecewise linear function that connects the highest observed outcome values (on the *Y*-axis) for each corresponding predictor value (on the *X*-axis). It is the most widely used and recommended technique in NCA due to its flexibility, intuitiveness, minimal assumptions about the shape of the ceiling line, and ease of application to dichotomous, discrete, and continuous variables, particularly those with fewer levels [8].

In addition to visually examining the *ceiling zone*, the primary indicator of necessity in NCA is the effect size (*d*). Unlike Cohen’s *d*, which assesses standardized mean differences between groups, NCA’s *d* is an effect size, which quantifies the strength of a necessity relationship by computing the ratio between the *ceiling zone* and the *scope*—that is, the degree to which the predictor is necessary for the outcome [8]. The NCA’s effect size is interpreted as follows: 0 < *d* < 0.10 indicates a small effect, 0.10 ≤ *d* < 0.30 indicates a medium effect, 0.30 ≤ *d* < 0.50 indicates a large effect, and *d* ≥ 0.50 indicates a very large effect [8]. Following recent guidelines [26], this study considered a predictor to be a necessary condition (i.e., necessity in kind or “X is necessary for Y”) only if it achieved an effect size of *d* ≥ 0.10 and an estimated *p* < 0.05. Data analysis was conducted using 10,000 resamples with approximate permutation methods. Bottleneck tables were produced for statistically significant necessary conditions, providing insights into the levels of necessary conditions required to achieve specific outcome levels (i.e., necessity in degree or “level x of X is necessary for level y of Y”). In this study, we aimed to assess whether lower self-esteem serves as a necessary condition for three distinct outcomes—*Drive for Thinness, Body Dissatisfaction,* and *Bulimia*—and if so, to identify the threshold value of self-esteem needed to reach subclinical levels of these behaviors [25]. All NCAs were performed using the R package “NCA”, version 4.0.1.

## Results

### Descriptive statistics

Descriptive statistics of the sample are reported in Table 1.

**Table 1:** Descriptive statistics

Variable	M	SD	Empirical range	Theoretical range
Rosenberg scale				
At baseline	28.23	4.84	15.00–38.00	10–40
EDI-2—drive for thinness				
At baseline	6.45	6.22	0.00–20.00	0 – 21
At 12 months	6.35	6.39	0.00–21.00	
EDI-2—body dissatisfaction				
At baseline	9.75	6.75	0.00–23.00	0–25
At 12 months	8.95	6.23	0.00–25.00	
EDI-2—Bulimia				
At baseline	1.64	2.73	0.00–15.00	0–21
At 12 months	1.24	2.14	0.00–10.00	

### Necessary condition analysis

#### Necessity in kind

**Fig. 1:**
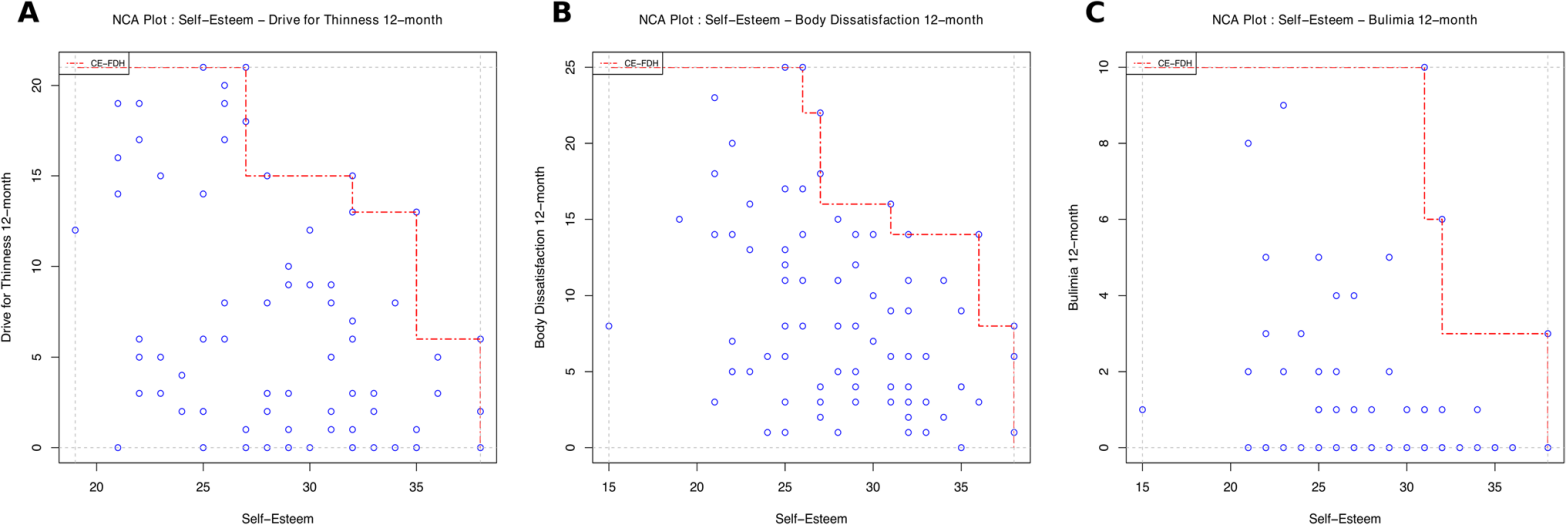
NCA scatterplots between baseline self-esteem, assessed using the Rosenberg Self-Esteem Scale as a predictor, and drive for thinness (*panel A*), body dissatisfaction (*panel B*), and Bulimia *(panel C)* assessed using the Eating Disorder Inventory-II, as outcomes at the 12-month follow-up

Lower levels of self-esteem at baseline were found to be a statistically significant necessary condition for higher levels of both *Drive for Thinness* after 12 months (*d* = 0.25, medium effect, *p* = 0.003, *n* = 75), and *Body Dissatisfaction* after 12 months (*d* = 0.22, medium effect, *p* = 0.003, *n* = 77) (Fig. 1, panels A and B). In contrast, self-esteem levels did not emerge as a statistically significant necessary condition for *Bulimia* after 12 months (*d* = 0.20, *p* = 0.415, *n* = 76; Fig. 1, panel C).

#### Necessity in degree

We then conducted a bottleneck analysis to determine the baseline self-esteem levels necessary to reach the identified threshold (95th percentile) for subclinical levels of *Drive for Thinness* and *Body Dissatisfaction* after 12 months. As shown in Table 2, achieving a score of 19 on the *Drive for Thinness* subscale required a baseline self-esteem score of 27 or lower. Likewise, reaching a score of 20 on the *Body Dissatisfaction* subscale required a baseline self-esteem score of 27 or lower (Table 2). These bottleneck thresholds represent the necessary conditions for the onset of subclinical restriction-oriented cognitions, as measured by *Drive for Thinness,* and dissatisfaction with the body, as measured by *Body Dissatisfaction,* over a 12-month period. The analysis also indicates that without meeting the baseline self-esteem thresholds, achieving subclinical levels of *Drive for Thinness* and *Body Dissatisfaction* after 12 months would not be possible.

**Table 2:** Bottleneck table of the identified significant necessary condition (self-esteem at baseline) for drive for thinness and body dissatisfaction (EDI-2) at 12 months

Outcome	Necessary condition	Outcome	Necessary condition
Drive for thinness (EDI-2 drive for thinness) after 12 months	Self-esteem (RSES) at baseline	Body dissatisfaction (EDI-2 body dissatisfaction) after 12 months	Self-esteem (RSES) at baseline
0	38	0	38
1	38	1	38
5	38	5	38
10	35	10	36
15	32	15	31
19 (95th percentile)	27	20 (95th percentile)	27

Furthermore, 42.67% of the sample met the necessary condition (i.e., a self-esteem score of 27 or lower) for developing high levels of *Drive for Thinness* at 12 months, and 45.45% met the necessary condition for *Body Dissatisfaction* at 12 months. However, only 8% of this group actually reached high levels of *Drive for Thinness* at follow-up, and 6.49% reached the threshold for high levels of *Body Dissatisfaction*. This indicates that, in addition to meeting the bottleneck value for low self-esteem, these individuals also reached or exceeded the 95th percentile on the *Drive for Thinness* and *Body Dissatisfaction* scales. As such, they are considered in this context to exhibit restriction-oriented cognitions and high discomfort with their own body, rather than merely a vulnerability to these conditions. In contrast, 57.33% and 54.55% of the sample did not meet the necessary condition and could be considered virtually immune to developing high levels of restriction-oriented cognitions and body dissatisfaction, respectively.

#### Sensitivity analysis

Finally, given the presence of missing data at baseline (self-esteem = 7%) and at follow-up (drive for thinness = 5%, bulimia = 2%, and body dissatisfaction = 4%), we evaluated the robustness of our results after imputing missing with multivariate imputation by chained equations (MICE). The results remained consistent for *Drive for Thinness* (*d* = 0.20, medium effect, *p* = 0.005, *n* = 84), *Bulimia* (*d* = 0.20, *p* = 0.435, *n* = 84), and *Body Dissatisfaction* (*d* = 0.22, medium effect, *p* = 0.002, *n* = 84).

## Discussion

Numerous probabilistic sufficient factors predicting the onset and persistence of ED symptoms have been identified [27, 28]. However, the corresponding necessary conditions remain largely unknown. In this study, we adopted a novel approach—NCA [8]—to complement traditional sufficiency-based methods by identifying potential necessary conditions for ED vulnerability. We focused on one of the most well-established risk factors in ED research: low self-esteem, examining its role in a sample of female adolescents, a group known to be at heightened risk for both ED symptoms and full-threshold disorders [21]. Specifically, we investigated *Drive for Thinness* and *Body Dissatisfaction*, which primarily capture cognitive–affective restrictive tendencies (e.g., preoccupation with weight gain, guilt or anxiety about eating high-calorie foods, and fear or shame about the body), as well as *Bulimia*, which reflects dysregulated eating behaviors (e.g., loss-of-control episodes, overeating in response to distress, and binge eating).

Pending future replication, our findings suggest that adolescent girls with high self-esteem may be virtually immune from developing subclinical levels of *Drive for Thinness* and *Body Dissatisfaction*. Both theoretical and empirical work indicate that global self-esteem is shaped by multiple domains, each contributing to overall self-evaluation [29]. Consistent with the centrality of physical appearance in adolescents’ self-concept and self-worth [30, 31], recent work shows a particularly strong link between self-esteem and body appearance in adolescent girls, a pattern not observed in boys (e.g., [32]). Taken together, these different streams of evidence suggest that it may be virtually impossible for girls with high global self-esteem to experience significant body-related distress or rigid beliefs about the need to lose substantial weight.

This interpretation aligns with Pennesi and Wade’s [17] systematic review of theoretical models of disordered eating, which emphasized self-esteem deficits as a core construct across multiple developmental and maintenance frameworks. Similarly, Fairburn’s transdiagnostic model [18, 32] highlights lower self-esteem as a key maintaining—and potentially predisposing—factor across EDs. More recently, Puttevils et al. [33] argued that low self-esteem, particularly when coupled with perfectionism, is central to the development and maintenance of anorexia-related symptoms, due to features, such as negative self-evaluation, cognitive rigidity [34], and excessive control over eating and body shape, which primarily reflect cognitive–affective rather than behavioral processes.

Our findings also resonate with the Tripartite Influence Model [35] and its integration with social comparison theory [36], which propose that sociocultural pressures foster thin-ideal internalization and appearance-based comparisons, thereby increasing body dissatisfaction and thinness-oriented cognitions. In this context, lower self-esteem may amplify these processes, widening the gap between adolescents’ actual and ideal selves and making self-worth more contingent on shape and weight. This mechanism may help explain why self-esteem emerged as a necessary condition for *Drive for Thinness* and *Body Dissatisfaction* in our analyses.

By contrast, self-esteem was not identified as a necessary condition for reporting subclinical levels of *Bulimia* after 12 months. In other words, girls with high global self-esteem may still experience dysregulated eating behaviors. On one hand, this is consistent with theoretical models suggesting that bulimic symptoms are more strongly linked to impulsivity, emotion dysregulation, and interpersonal difficulties rather than self-related cognitions [33, 37]. On the other hand, our results indicate that self-related cognitions (i.e., self-esteem) exert a constraining effect more specifically on the cognitive–affective restrictive tendencies (e.g., *Drive for Thinness* and *Body Dissatisfaction*) rather than on behavioral manifestations (i.e., *Bulimia*). Finally, it is also possible that the modest internal consistency of the scale—although consistent with previous literature [25]—may have limited our ability to detect a significant necessary condition effect.

Importantly, our bottleneck analysis indicated that not only low, but also average levels of self-esteem may be necessary for the development of high levels of restriction-oriented cognitions. Interestingly, only individuals with above-average self-esteem were virtually immune to experiencing high levels of *Drive for Thinness* and *Body Dissatisfaction*. On one hand, this finding parallels prior NCA research showing that average levels of intolerance of uncertainty are necessary for subclinical anxiety symptoms in adolescents [14]. On the other hand, a substantial portion of adolescents in our sample met the required necessary condition level without developing subclinical symptoms. This suggests that multiple necessary conditions (beyond those tested here) likely operate together, alongside probabilistic sufficient factors, to produce subclinical ED symptoms. Future theoretical work should, therefore, integrate both classes of causes—probabilistic and necessary—into comprehensive models of ED development (i.e., embedded theory [7]).

From a clinical perspective, the NCA framework may advance prevention efforts. By distinguishing individuals who are at genuine risk from those who are virtually immune, prevention strategies could move beyond universal programs—which may carry iatrogenic risks [38]—toward more selective, targeted interventions. Moreover, identifying necessary conditions can guide clinicians in addressing key mechanisms that must be present for ED symptoms to develop or persist. For instance, recent evidence shows the importance of specifically targeting self-esteem deficits in inpatients with anorexia [39]. Furthermore, unlike probabilistic models, which cannot be directly translated from population to individual cases, the deterministic logic of NCA allows for the identification of “a perfect predictor of the absence of diseases for nearly all people” ([9], p. 1), thereby offering insights for personalized interventions.

Future research should investigate necessary conditions across multiple domains and levels of analysis. It will be critical to examine whether conditions identified in non-clinical samples also apply to individuals with full-blown EDs, and whether factors such as perfectionism, emotional dysregulation, or impulsivity may also function as necessary conditions. Multi-level investigations spanning genetic, neurobiological, cognitive, emotional, social, and personality-related domains are needed. Finally, given that only ~ 50% of ED patients achieve full remission after standard treatments [40], NCA may help identify the necessary conditions for treatment success, highlighting which patients are unlikely to benefit from conventional therapies and may require alternative interventions.

Both strengths and weaknesses can be pointed out. A key strength of this study is the application of NCA, a novel methodological approach in the field of ED research. Departing from traditional sufficiency-based models, NCA provides an alternative perspective by identifying conditions that must be present for symptoms to emerge. By applying this approach to a well-established risk factor—low self-esteem—the study showed its necessity only for the development of restriction-oriented cognitions (i.e., *Drive for Thinness*) and body dissatisfaction in female adolescents, a population at elevated risk for EDs. These findings offer important theoretical and clinical insights, particularly for early detection and the development of targeted prevention strategies.

Nonetheless, several limitations should be acknowledged. First, the sample consisted exclusively of female adolescents, which restricts the generalizability of the findings to male or clinical populations. Future research should more extensively examine the sociodemographic characteristics of adolescents with and without the conditions associated with ED symptoms. Second, the reliance on self-report measures without diagnostic assessment limits the ability to establish the presence of clinically significant EDs. Third, although the sample size was adequate for NCA, larger and more diverse samples are needed to replicate and extend these findings. Finally, longer follow-up periods would provide valuable insights into the enduring constraining effects of self-esteem on the development of ED symptoms.

In conclusion, this study provides preliminary evidence supporting the utility of focusing on necessary conditions in ED research. By complementing traditional sufficiency-based approaches, the NCA framework has the potential to broaden our understanding of the mechanisms, developmental trajectories, and clinical pathways underlying EDs.

## What is already known on this subject?

Previous research, grounded in sufficiency logic, has identified low self-esteem as a key probabilistic risk factor in the development and maintenance of ED symptoms [15, 16], particularly in relation to restriction-oriented cognitions and body dissatisfaction [33]. However, its potential role as a necessary condition remains largely unexplored, limiting our understanding of both its critical contribution to the onset of ED symptoms when present and its protective function when absent.

## What this study adds?

The main contribution of this study is the introduction of NCA as a novel method for identifying necessary conditions for the development of ED symptoms in adolescents after a 12-month period. This approach has been rarely applied in clinical psychology, and even less so in ED research. Focusing on low self-esteem—a well-established probabilistic risk factor—the study reveals that it may also serve as a necessary condition for the emergence of restriction-oriented cognitions and body dissatisfaction, though not for dysregulated eating behaviors. Notably, only adolescents with above-average self-esteem appeared protected from developing high levels of *Drive for Thinness* and *Body Dissatisfaction*. These findings underscore the theoretical and clinical importance of integrating necessary conditions into existing models of EDs, offering new opportunities for more targeted and personalized prevention strategies.

## Data Availability

The data set analyzed during the current study is available from the corresponding author on reasonable request.
